# Supplementary material for: Cloning of Metalloproteinase 17 Genes from Oriental Giant Jellyfish Nemopilema nomurai (Scyphozoa: Rhizostomeae)
Source: Toxins (Basel). 2022 Jul 29;14(8):519. doi: 10.3390/toxins14080519 (PMC9414644; doi:10.3390/toxins14080519)
Supplement: Supplementary file 1 [file toxins-14-00519-s001.zip › toxins-1821380-supplementary.pdf]

```
JVMP17-1    -------------WNKQKITWR-----VEN------FGNDGLSPERVKMVFENALGKWSG    36
JVMP17-2    -------------WNKRNLTWA-----IAN------SNNDNIKDEDVKETILNALKKWSA    36
A8QL59      SNITVVRMRIFEILNYVNLYYKILNIHVVLIGLEVWSDEDKILINGSSELTVRSFAAWRH    60
ABN72537    RNKPIIKRRVYELVNILNTILRRLNFHIALIGLEIWSKRDKINVQSDVKATLKSFGKWRE    60
Q90ZI3      RNLTTLRTRMFDTVNIVNQILQRINIHVALIGIEIWSKEDKIIVQSVPDVTLKLFATWRE    60
Q8AWI5      RNLTTVRTRMYDIVNVINVIYQRMNIHVALVGLEIWSNKDKFILRSAADVTLKLFATWRE    60
Q9DGB9      RNLTAVRTRMYDIVNVITPIYHRMNIHVALVGLEIWSNTDKIIVQSSADVTLDLFAKWRA    60
O42138      DNLDKIKTRIFEIVNTMNEMFIPLNIRVALICLEIWSDKDKFNMTSAANVTSISFRNWRA    60
ABG26979    RKLTEVKTWVYEIVNTLNEIYRYLYIRVALVGLEIWSNGDLSNVTLSADDTLDSFGEWRK    60
Q9w6M5      GDLDKIKTKMYEAANNMNEMYRYMFFRVVMVGLIIWTEEDKITVKPDVDYTLNAFAEWRK    60
C9E1S0      GDLDKIKTRMYEIVNTVNEIYRYMYIHVALVGLEIWSNEDKITVKPEAGYTLNAFGEWRK    60
C9E1R8      GDLDKIKTRMYEIVNTVNEIYRYMYIHVALVGLEIWSNEDKITVKPEAGYTLNAFGEWRK    60
O93523.2    GDLDKIKARMYELANIVNEILRYLYMHAALVGLEIWSNGDKITVKPDVDYTLNSFAEWRK    60
Q8QG88      GDLDKIKARMYELANIVNEILRYLYMHAALVGLEIWSNGDKITVKPDVDYTLNSFAEWRK    60
                          *  .                     *              :  *

JVMP17-1    VTNIEFEEIKTGEPDIWFRFVRGAHGDPYP-----FRTPGSTVLAHAFYPFDSRTPLSGD    91
JVMP17-2    ITEIDFTEDK-NNPDIWIRFTRKYHNDASP-----FDGRGGE-LAHAFYPAN--DELSGD    87
A8QL59      SDL-----------------LKHKRNDNAQLLTGIHFDKRV---LGIAFIGGMCNNFTSVG    101
ABN72537    KKL-----------------LPRKRNDNAQLLTRIDFNGNT---VGLAALGSLCSVKYSVA    101
Q90ZI3      SVL-----------------LKRKNHDNAHLLTGINFNGPT---AGLAYLGGICKPMYSAG    101
Q8AWI5      TDL-----------------LKRKSHDNAQLLTGINFNGPT---AGLGYLGGICNPMYSAG    101
Q9DGB9      TDL-----------------LSRKSHDNAQLLTGINFNGPT---AGLGYLGGICNTMYSAG    101
O42138      TDL-----------------LKRKSHDNAQLLTVIDFDGPT---IGKAYMASMCDPKRSVG    101
ABG26979    RDL-----------------LRRKSHGNAQLLTAIDFNGTT---IGLAHVASMCELKRSTG    101
Q9w6M5      TYL-----------------LAEKKHDNAQLITGIDFRGSI---IGYAYIGSMCHPKRSVG    101
C9E1S0      TDL-----------------LTRKKHDNAQLLTAIDLD-RV---IGLAYVGSMCHPKRSTG    100
C9E1R8      TDL-----------------LTRKKHDNAQLLTAIDLD-RV---IGLAYVGSMCHPKRSTG    100
O93523.2    TDL-----------------LTRKKHDNAQLLTAIDFNGPT---IGYAYIGSMCHPKRSVA    101
Q8QG88      TDL-----------------LTRKKHDNAQLLTAIDFNGPT---IGYAYIGSMCHPKRSVA    101
                               : .  :.:        :        . .          *

JVMP17-1    VHFNDDKVFTIESPSGKDLLWISVHELGHSIGLDHSDVKS-------SIMYPYYRGYPGL    144
JVMP17-2    VHYDDDEVFSIKSSQGRDLLWITVHEVGHSLGLDHSGTQG-------SVMNAFYRETKGL    140
A8QL59      -------AIQDNSIHAVLIAATMTHELGHNLGMNHDTDSCT-CNTGPCIMKAALNFKPPY    153
ABN72537    -------VIQDYSKRTSMVASTMAHEMGHNLGINHDRASCTSCGSNKCIMATKRT-KPAS    153
Q90ZI3      -------IVQDHNKIHHLVAIAMAHEMGHNLGMDHDKDTCT-CRAKACVMAGTLSCDASY    153
Q8AWI5      -------IVQDHNKIHHLVAIAMAHEMGHNLGIDHDKDTCT-CGAKSCVMAGTLSCEASY    153
Q9DGB9      -------IVQDHSKIHHLVAIAMAHEMGHNLGMDHDKDTCT-CGTRPCVMAGALSCEASF    153
O42138      -------IIQDHSTINLMMAVTMAHEMGHNLGMDHDEKYCT-CGAKSCVMAKALSRQPSK    153
ABG26979    -------IVQDHSPINLLVAVTMAHEMGHNLGIRHDKKYCT-CGGYSCIMSAVLSHQASK    153
Q9w6M5      -------IIQDYSPINLVLAVIMAHEMGHNLGIHHDDGYCY-CGGYPCIMGPSISPEPSK    153
C9E1S0      -------IIQDYSEINLVVAVIMAHEMGHNLGINHDSGYCS-CGDYACIMRPEISPEPST    152
C9E1R8      -------IIQDYSEINLVVAVIMAHEMGHNLGINHDSGYCS-CGDYACIMRPEISPEPST    152
O93523.2    -------IVEDYSPINLVVAVIMAHEMGHNLGIHHDTDFCS-CGDYPCIMGPTISNEPSK    153
Q8QG88      -------IVEDYSPINLVVAVIMAHEMGHNLGIHHDTDFCS-CGDYPCIMGPTISNEPSK    153
                    .   .     :    .**:**.:*: *.           .:*

JVMP17-1    DFELTPDDV-------RGAQAL--------------YG-    161
JVMP17-2    DFNLHADDI-------KGAQSL--------------YG-    157
A8QL59      EFSSCSYWDFQNYIMTKSAQCILNDPLTTDIVPTAICGN    192
ABN72537    RFSSCSVREHQRYLLRDRPQCILNKPLITDIVAPAICGN    192
Q90ZI3      LFSDCSRQEHRAFLIKNMPQCILKKPLKTDVVSPPVCGN    192
Q8AWI5      LFSDCSRKEHQAFLIKDMPQCILKKPLKTDVVSPPVCGN    192
Q9DGB9      LFSDCSQKDHREFLIKNMPQCILKKPLKTDVVSPAVCGN    192
O42138      LFSNCSQEDYRKYLIKRRPKCILNEPNGTDIVSPPVCGN    192
ABG26979    YFSNCSYNQYWNYINFYKPQCILNEPLRTDIVSPPVCGN    192
Q9w6M5      FFSNCSYIQCWDFIMNHNPECIDNEPLGTDIISPPLCGN    192
```

```
C9E1S0        FFSNCSYFECWDFIMNHNPECILNEPLGTDIISPPVCGN        191
C9E1R8        FFSNCSYFECWDFIMNHNPECILNEPLGTDIISPPVCGN        191
O93523.2      FFSNCSYIQCWDFIMKENPQCILNEPLGTDIVSPPVCGN        192
Q8QG88        FFSNCSYIQCWDFIMKENPQCILNEPLGTDIVSPPVCGN        192
                 *.                :.:            *
```

Supplementary Figure S1. Alignment analysis of JVMP17-1 and 17-2 M domains compared to that of other SVMPs M domain. A8QL59 : *Naja atra*, ABN72537 : *Bungarus multicinctus*, Q90ZI3 : *Protobothrops flavoviridis*, Q8AWI5 : *Gloydius halys*, Q9DGB9 : *Crotalus atrox*, O42138 : *Agkistrodon contortrix laticinctus*, ABG26979 : *Sistrurus catenatus edwardsi*, Q9w6M5 : *Deinagkistrodono acutus*, C9E1S0 : *Agkistrodon piscivorus leucostoma*, C9E1R8 : *Crotalus viridis*, O93523.2 : *Bothrops jararaca*, Q8QG88 : *Bothrops insularis*. Identical, similar, and weakly similar amino acids are indicated by asterisks, colons and dots, respectively.

Supplementary Table S1. Comparison of *N. nomurai* JVMP 17-1 and JVMP 17-2 deduced amino acid sequence with a BLAST analysis

	Scientific name	Description	identity (%)	Genbank No.
JVMP17-1	*Hydra vulgaris*	MMP-24	42.18	XP_012564333.2
	Acropora digitifera	MMP-24-like	40.16	XP_015771447.1
	Orbicella faveolata	MMP-24-like	39.84	XP_020618480.1
	Nematostella vectensis	MMP-17	37.38	EDO48606.1
	Apis cerana	MMP-14 isoform	37.27	XP_016905171.1
	Exaiptasia diaphana	MMP-17-like	36.33	XP_020891959.1
	Bufo gargarizans	MMP-17-like	35.49	XP_044130425.1
JVMP17-2	*Hydra vulgaris*	MMP-24	37.72	XP_012564333.2
	Acropora millepora	MMP-24-like	37.25	XP_029201938.2
	Orbicella faveolata	MMP-24-like	39.58	XP_020618480.1
	Xenopus laevis	MMP-17	35.98	XP_041433135.1
